# Sensory Disturbance of the Lower Extremity after Sural Artery Flap Elevation

**DOI:** 10.1155/2021/8886063

**Published:** 2021-02-13

**Authors:** Kaoru Tada, Seigo Suganuma, Mika Nakada, Masashi Matsuta, Atsuro Murai, Hiroyuki Tsuchiya

**Affiliations:** Department of Orthopaedic Surgery, Graduate School of Medical Science, Kanazawa University, 13-1 Takaramachi, Kanazawa 920-8641, Japan

## Abstract

**Purpose:**

Elevation of the sural artery flap with the sural nerve is associated with donor-site morbidities, such as postoperative sensory disturbance of the lower extremity. We evaluated the sensory disturbance of the lower extremity after elevation of the sural artery flap.

**Methods:**

This study included 7 patients who underwent surgery using the sural artery flap. The sensory disturbances immediately after surgery and at present were evaluated on a 10-point scale. The influences of surgery on activities of daily living and patient satisfaction were also evaluated.

**Results:**

The sensory disturbance was 4.48 immediately after surgery and 2.24 presently, and the difference between the timepoints was not statistically significant. The influence of surgery on activities of daily living was 2.30, and the patient satisfaction was 7.90.

**Conclusion:**

It may be necessary to consider the sural artery flap, which does not include the sural nerve, to avoid unnecessary complications. When it is unavoidable to use the sural artery flap, including the sural nerve, it is important to thoroughly inform patients beforehand about the postsurgery sensory disturbance in the lower extremities.

## 1. Introduction

The sural artery flap is generally elevated with the sural nerve and its accompanying artery, as well as the lesser saphenous vein and its accompanying artery. Since this flap is a pedicled flap that does not require vascular anastomosis and is easy to elevate, it is widely used to treat soft tissue defects in the lower limbs and feet; to date, there are numerous studies that report the use of the sural artery flap [1–7]. However, because the sural artery flap includes the sural nerve, sensory disturbance of the lower extremity (especially on the lateral part of the foot) often occurs postoperatively. Many studies on the sural artery flap focus on the survival rate of the flap, and there are only a few studies that have evaluated the extent and course of sensory disturbance of the lower extremity [8]. In recent reviews, sensory disturbance due to sacrifice of the sural nerve has not been considered as a major complication [3, 7].

In this case report, we investigated the sensory disturbance of the lower extremity in patients who were treated with the sural artery flap for soft tissue defects.

## 2. Methods

### 2.1. Patients

Between 2008 and 2018, 17 patients underwent reconstruction for soft tissue defects of the lower extremity in our hospital using the sural artery flap. 10 of 17 patients had a different address and could not be located. Of the remaining 7 patients (5 males and 2 females) who agreed to participate in the study were included and completed the questionnaire. In all patients, we elevated the sural artery flap, which included the sural nerve and its accompanying artery, as well as the small saphenous vein and its accompanying artery.

### 2.2. Evaluations

Evaluations were conducted using a questionnaire with a 10-point scale. Evaluation items included the sensory disturbance of the lower extremity (0 = no disturbance, 10 = extreme disturbance) immediately after surgery and at present, the current influence of surgery on activities of daily living (ADL) (0 = no trouble, 10 = troubling), and satisfaction with the reconstruction surgery (0 = unsatisfied, 10 = completely satisfied).

### 2.3. Statistical Analyses

Patient characteristics, including age and time since surgery, and questionnaire data, including the sensory disturbance and all other evaluations, were expressed as median (interquartile range (25^th^ and 75^th^ percentile)). The sensory disturbance between the two timepoints was compared using the Wilcoxon signed-rank test. *P* < 0.05 was considered statistically significant, and all *P* values were two-tailed. Statistical analyses were performed using SPSS software, version 22.0 (IBM Corp, NY, US).

### 2.4. Ethical Considerations

This retrospective research was approved by the institutional review board of our institution. All procedures were conducted in accordance with the ethical standards of the Committee on Human Experimentation in our institution and the Helsinki Declaration of 1975. Written informed consent was obtained from each patient prior to inclusion in the study.

## 3. Results

The median age at the time of surgery was 47.0 (21.5, 63.5) years, and the median time since surgery was 7.0 (5.5, 8.5) years. Soft tissue defects occurred due to a tumor in 4 cases and trauma in 3 cases. The site of the soft tissue defect was the knee joint in 1 patient, the lower leg in 2 patients, the medial condyle in 3 patients, and the heel in 1 patient. 5 flaps were distally based, while 2 were proximally based.

The sensory disturbance was 4.48 (2.85, 6.46) immediately after surgery and is 2.24 (1.33, 4.61) presently. Symptoms tended to improve, but the difference between the timepoints was not statistically significant (*P*=0.17). The influence of surgery on ADL was 2.30 (1.03, 3.34), while the patient satisfaction with reconstruction surgery was 7.90 (6.01, 9.05). In one case where a proximally based flap was used, hyperesthesia at the tip of the flap and radiation pain to the lower extremity occurred, which was thought to be due to stump neuroma.

## 4. Discussion

As a result of this study, sensory disturbance after using a sural artery flap was proven to be mild but remained, and no significant improvement was observed even 7 years after the surgery. The sural artery flap does not necessarily need to include the sural nerve, so it may be necessary to consider the sural artery flap, which does not include the sural nerve, to avoid unnecessary complications.

Since the development of the sural artery flap, it remains debatable whether the sural nerve should be included in the flap. Donski et al. developed the prototype of the sural artery flap, defining it as a “distally based fasciocutaneous flap from the sural region,” and reporting that the sural nerve should not be included in the flap, if possible [9]. In addition, Masquelet et al. defined the sural artery flap as a “skin island flap supplied by the vascular axis of the sensitive superficial nerves.” They indicated that the flap includes the sural nerve and reported that one problem of the flap was that it sacrificed the sensory nerve [10]. Later, the results of an anatomical study by Aoki et al. suggested that the accompanying artery around the sural nerve seems to have the most significant impact on flap survival and that the sural nerve has fewer intrinsic vessels than its accompanying artery [11]. Their sural artery flap included the accompanying artery, which was detached from the sural nerve, and did not include the sural nerve itself. However, detachment of the sural nerve and its accompanying artery is complicated and time-consuming, especially in the proximal part of the lower leg where the nerve and artery run below the deep fascia [2, 12]. Therefore, many studies have selected the sural artery flap that includes the sural nerve [1, 2, 4–6]. Donor-site morbidities caused by sacrifice of the sural nerve have rarely been considered in previous studies. Cheema et al. reported that “the loss of sensation of the foot has not been a significant clinical problem in our patients” [1].

However, the sensory disturbance after using a sural artery flap was proven to be mild but remained in this study. Therefore, flap elevation without inclusion of the sural nerve should be carefully considered so that it does not affect flap survival. The methods of Donski and Fogdestam [9] and the method of Nakajima et al. [13], which involves the veno-accompanying artery fasciocutaneous flap and uses the accompanying artery of the lesser saphenous vein as a feeding vessel without including the sural nerve and its accompanying artery, are considered effective. Furthermore, the sural nerve splitting method recently reported by Kim et al. [8], which preserves the medial sural cutaneous nerve, is also a reasonable method. If including the sural nerve is unavoidable, the nerve should be sharply dissected (so that a stump neuroma will not form) and the nerve stump should be embedded in the adipose tissue.

Because the patients who were treated with the sural artery flap originally had tumors and trauma of the lower extremity, it is considered that the surgery was invasive. However, unlike pain, sensory disturbance is not a symptom caused by surgical invasion itself, so it is thought that the sacrifice of the sural nerve is the main cause of sensory disturbance.

We believe that the results were partially affected by insufficient communication about sensory disturbance prior to surgery. When using the sural nerve autograft for reconstruction of the peripheral nerve defect, doctors commonly inform patients of the expectations regarding recovery and sensory disturbance, which is due to the sacrifice of the healthy sural nerve [14]. On the other hand, when using the sural artery flap for soft tissue defects in the lower extremity, explanations about flap survival and functional prognosis of the lower extremity are prioritized, and information regarding sensory disturbance of the lower extremity after flap elevation may be insufficient. As a result, sensory disturbance is often considered as an unexpected complication by these patients leading to poor outcome. In particular, a recent systematic review indicated that preoperative patient education reduced postoperative patient complaints [15]. In order to reduce the complaints after elevation of the sural artery flap, the best thing is to not include the sural nerve in the flap. However, if including the sural nerve is unavoidable, it is important to share sufficient information beforehand regarding the postsurgery sensory disturbance in the lower extremities with the patients.

The limitations of this study include the small sample size and the presence of sampling and recall biases, especially for the sensory disturbance evaluated immediately after surgery. Therefore, caution is needed when interpreting our results. Also, because this study is designed as a questionnaire survey, it was not possible to evaluate the patients using the Semmes–Weinstein monofilament test or the two-point discrimination test. In future, we would like to compare these results with the results of the sural artery flap that does not include the sural nerve.

## 5. Conclusions

The sensory disturbance after using a sural artery flap was proven to be mild but remained, and no significant improvement was observed even 7 years after the surgery. It may be necessary to consider the sural artery flap, which does not include the sural nerve, to avoid unnecessary complications. When it is unavoidable to use the sural artery flap, including the sural nerve, it is important to thoroughly inform patients beforehand about the postsurgery sensory disturbance in the lower extremities.

## Data Availability

The data used to support the findings of this study are available from the corresponding author upon request.
